# RNA m^5^C methylation: a potential modulator of innate immune pathways in hepatocellular carcinoma

**DOI:** 10.3389/fimmu.2024.1362159

**Published:** 2024-05-13

**Authors:** Sun Meng, Bai Jiangtao, Wang Haisong, Li Mei, Zhou Long, Li Shanfeng

**Affiliations:** Department of Interventional Vascular Surgery, Affiliated Hospital of Hebei University, Baoding, China

**Keywords:** RNA m5C methylation, TLR, cGAS-STING, RIG-I, hepatocellular carcinoma

## Abstract

RNA 5-methylcytosine (m^5^C) methylation plays a crucial role in hepatocellular carcinoma (HCC). As reported, aberrant m^5^C methylation is closely associated with the progression, therapeutic efficacy, and prognosis of HCC. The innate immune system functions as the primary defense mechanism in the body against pathogenic infections and tumors since it can activate innate immune pathways through pattern recognition receptors to exert anti-infection and anti-tumor effects. Recently, m^5^C methylation has been demonstrated to affect the activation of innate immune pathways including TLR, cGAS-STING, and RIG-I pathways by modulating RNA function, unveiling new mechanisms underlying the regulation of innate immune responses by tumor cells. However, research on m^5^C methylation and its interplay with innate immune pathways is still in its infancy. Therefore, this review details the biological significance of RNA m^5^C methylation in HCC and discusses its potential regulatory relationship with TLR, cGAS-STING, and RIG-I pathways, thereby providing fresh insights into the role of RNA methylation in the innate immune mechanisms and treatment of HCC.

## 1 Introduction

Hepatocellular carcinoma (HCC) is renowned as the “Monarch of Malignancies” due to its high malignancy degree and dismal prognosis. Additionally, the clinical diagnosis and management of HCC are riddled with formidable challenges, such as difficult early detection, high recurrence and metastasis rates, lack of targeted therapies, and limited innovative drugs and treatment modalities (1, 2). Hence, it is pressing and pivotal to comprehensively investigate the molecular mechanisms of HCC.

As the frontline defense in the body, the innate immune system encompasses a diverse array of myeloid lineage cells, including dendritic cells (DCs), monocytes, and macrophages. Because these cells can function as professional antigen-presenting cells (APCs) or innate lymphocytes such as natural killer (NK) cells, they can swiftly detect microbial proteins or nucleic acid molecules on tumor cells by relying on pattern recognition receptors (PRRs) and other cell surface receptors, therefore orchestrating downstream immune responses (3, 4). For immune surveillance in HCC, various PRRs, such as cyclic guanosine monophosphate-adenosine monophosphate synthase (cGAS)-stimulator of interferon genes (STING) (5), retinoic acid-inducible gene I (RIG-I) (6), and Toll-like receptors (TLRs) (7), can favor the transcription of pro-inflammatory genes via pathways involving interferon regulatory factor 3 (IRF-3) and nuclear factor kappa B (NF-κB), inducing the production of interferons (IFNs), cytokines, and chemokines and ultimately potentiating cytotoxic anti-tumor responses mediated by effector T and NK cells. Nevertheless, little is known regarding mechanisms underlying the evasion of tumor cells from innate immune surveillance.

RNA methylation plays a critical role in modulating immune responses, among which 5-methylcytosine (m^5^C) methylation is an emerging RNA modification mechanism with the function of governing RNA stability, translational regulation, and various cellular functions such as proliferation and differentiation (8, 9). The constant expansion of the m^5^C methylase family has unveiled the critical involvement of m^5^C methylation in diverse diseases, prominently in cancers (10). m^5^C methylases have become a focal point in research on disease markers and potential therapeutic targets since their aberrant expression has been intricately associated with the development, progression, and treatment responses of tumors (11–13).

Several studies uncovered that m^5^C methylation affected the growth and immune evasion of tumors (14–16). Yet, it is still in the nascent stage to investigate the role of RNA m^5^C methylation in innate immunity against HCC. Accordingly, this review comprehensively analyzes the involvement of m^5^C methylation in the onset and progression of HCC and its mechanisms in the innate immune responses of HCC, thus furnishing a novel theoretical framework for the treatment of HCC.

## 2 Overview of RNA m^5^C methylation

### 2.1 Detection methods of m^5^C methylation sites

**Figure 1:**
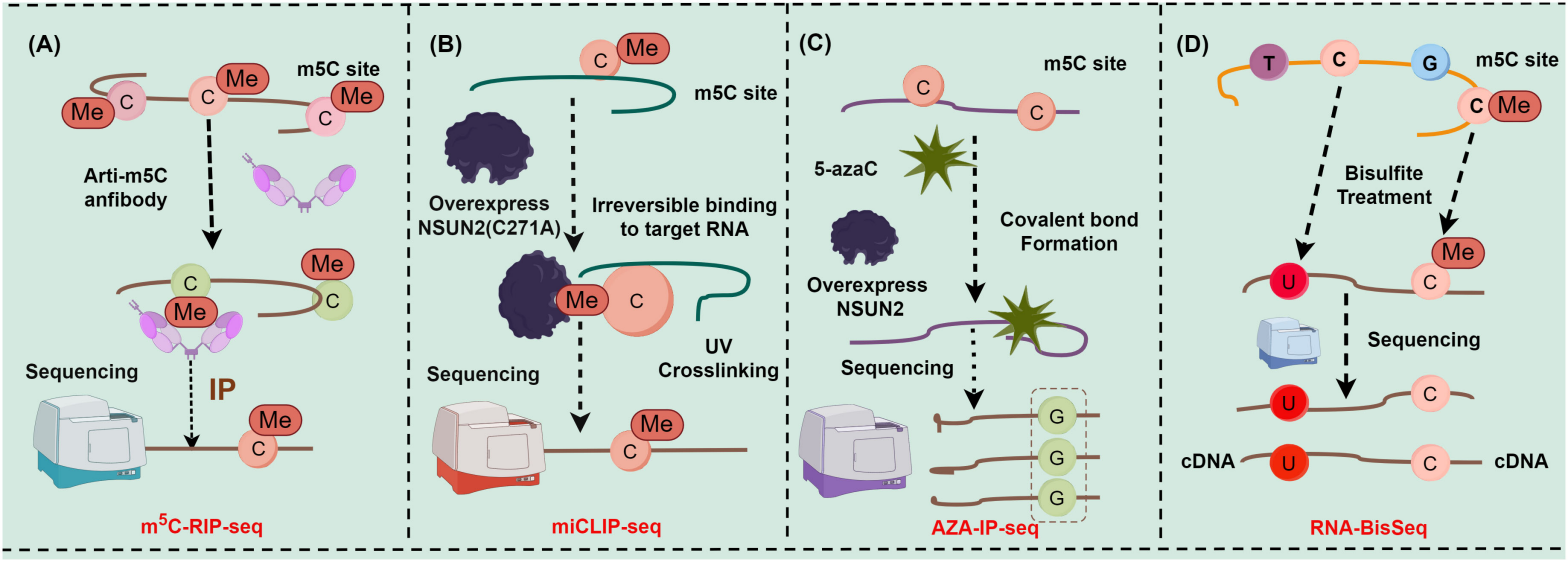
Major detection methods of m^5^C methylation sites (figures by Figdraw). **(A)** Detection steps for m^5^C-RIP-seq; **(B)** Detection steps for miCLIP-seq; **(C)** Detection steps for AZA-IP-seq; **(D)** Detection steps for RNA-BisSeq.

As a crucial reversible epigenetic modification, m^5^C methylation is extensively present in various RNA types, encompassing mRNA, rRNA, tRNA, long non-coding RNA, circular RNA, microRNA, and vtRNA. Methylation at diverse sites assumes a pivotal role in mediating the fate of RNA, including the stability, extranuclear transport, and transcription of RNA (13, 17). Intriguingly, m^5^C methylation sites on RNA can be detected and localized more precisely with the advent of cutting-edge high-throughput sequencing technologies, such as RNA bisulfite sequencing (RNA-BisSeq) (18, 19), 5-azacytidine-mediated RNA immunoprecipitation sequencing (AZA-IP-seq) (20), methylation individual nucleotide resolution crosslinking (miCLIP-seq) (21, 22), and immunoprecipitation sequencing (m^5^C-RIP-seq) (18, 23). For instance, m^5^C-RIP-seq can enrich m^5^C-containing RNA fragments for sequencing via antibodies specifically targeting m^5^C methylation sites^15^, (23) (**Figure 1A**). Although m^5^C-RIP-seq can effectively identify highly methylated RNA fragments, it has limited resolution of peaks and much lower rates and fails to obtain the precise location and absolute level of m^5^C in the transcriptome compared with other sequencing methods with single-nucleotide resolution since the RNA fragments enriched by antibodies are about 100−150 nt in length. In addition, m^5^C-RIP-seq cannot identify methylation on low-abundance mRNAs because the statistical analysis is relatively insensitive to low-coverage regions (24). miCLIP-seq can capture NOL1/NOP2/SUN domain 2 (NSUN2)-targeted RNA fragments through antibodies against NSUN2. Hussain et al. utilized miCLIP-seq technology to localize the m^5^C methylation site. Specifically, through ultraviolet light, overexpressed mutant NSUN2 (C271A) was cross-linked with RNA fragments to form RNA-protein complexes containing robust covalent bonds, and truncation or mutations were induced during reverse transcription polymerase chain reaction (RT-PCR) and then identified with sequencing data to map the m^5^C methylation site (21, 22) (**Figure 1B**). However, it has been also reported that because NSUN2 catalyzes m^5^C methylation in only a subset of RNAs, a comprehensive transcriptomic profile cannot be obtained with antibodies against a single RNA m^5^C methyltransferase. Furthermore, miCLIP-seq requires overexpression of NSUN2 with mutations in the m^5^C release domain, which creates an abnormal intracellular environment and limits the application of this technology to cells cultured *in vitro (* 25). Furthermore, AZA-IP-seq can detect m^5^C sites in RNA by replacing cytosine with 5-azacytidine (20) (**Figure 1C**). Similar to miCLIP-seq, AZA-IP-seq also can only detect m^5^C sites in a subset of RNAs that are catalyzed by a single RNA m^5^C methyltransferase, with an incomplete transcriptomic map. Additionally, the detection results of AZA-IP-seq depend on the efficiency of 5-azaC introduction, which results in reduced sensitivity to m^5^C sites in low-abundance RNA (20). Although the use of AZA-IP-seq is limited to the detection of toxic and specific RNA subpopulations, this technology remains valuable for research on m^5^C methylation. RNA-BisSeq, additionally, is a widely applied detection method for RNA m^5^C methylation (18, 19) (**Figure 1D**), which converts cytosine to uracil through bisulfite treatment, followed by PCR detection of unconverted cytosine to determine the m^5^C site. Despite the advantages of single-nucleotide resolution and methylation level analysis, which avoids some of the drawbacks of other techniques, RNA-BisSeq fails to distinguish between unconverted cytosines derived from m^5^C and 5-hydroxymethylcytosine (hm^5^C) and may encounter recognition errors owing to the double-stranded structure. Conclusively, the evolution of these technologies tremendously advances the understanding of the pivotal role of RNA modifications in gene expression regulation, disease progression, and cellular functions.

### 2.2 Molecular mechanisms underlying m^5^C methylation

**Figure 2:**
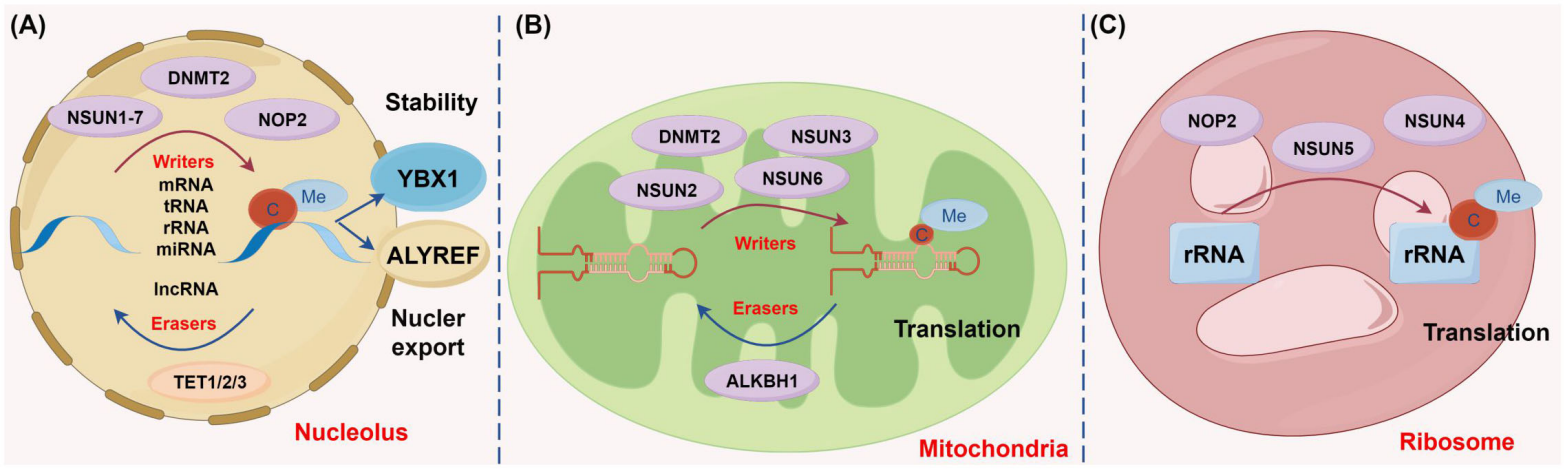
Diverse biological functions of m^5^C methylation regulators across different cellular compartments (figure by Figdraw). **(A)** In the nucleus, genes such as NSUN1-7, DNMT2, and NOP2 function as the catalysis enzymes of m^5^C methylation to modulate methylation modifications on multiple RNA types including mRNA, rRNA, tRNA, miRNA, and lncRNA. Conversely, the demethylases TET1, TET2, and TET3 erase m^5^C methylation of these RNAs. The m^5^C methylation recognition protein YBX1 stabilizes RNA, whereas ALYREF facilitates the nuclear export of RNA. **(B)** In mitochondria, m^5^C methylases, including NSUN2, NSUN3, NSUN6, and DNMT2, and the demethylase ALKBH1 collaboratively regulate mitochondrial tRNA stability and participate in protein translation. **(C)** In ribosomes, the methylases NOP2, NSUN4, and NSUN5 promote rRNA stability and are involved in protein translation.

First discovered in 1925, m^5^C methylation is currently known to be present on various RNA molecules in multiple organelles, including nuclei (**Figure 2A**), mitochondria (**Figure 2B**), and ribosomes (**Figure 2C**) (26–28). There are over 90,000 m^5^C sites detected in the human genome, and m^5^C methylation is preferentially deposited at the translation start site, near the 3’-untranslated region, and near the Argonaute binding region of mRNAs (29).

Mechanistically, RNA m^5^C methylation primarily involves three categories of effectors, namely methyltransferases, demethyltransferases, and m^5^C readers.

(1) Methyltransferases: m^5^C methylation is typically catalyzed by methyltransferases, such as NSUN family members (NSUN1-7) and DNA methyltransferase homolog 2/tRNA-aspartic acid methyltransferase 1 (DNMT2/TRDMT1). During m^5^C methylation, the cysteine residue in the methyltransferase forms a covalent intermediate with the cytosine in the target RNA, which enables the C atom at position C5 to become nucleophilic and to bind to the methyl group of S-adenosyl methionine, contributing to the transfer of the methyl group (27). (2) Demethyltransferases: m^5^C demethylation is primarily achieved by the ten-eleven translocation (TET) family (TET1-3) and ALKBH1. Specifically, TET enzymes, as α-ketoglutarate- and Fe^2+^-dependent dioxygenases, are responsible for catalyzing m^5^C demethylation to generate hm^5^C. Recent studies have unveiled that TET overexpression substantially enhances RNA hm^5^C levels, highlighting that members in the TET family may also function as RNA demethylases to remove m^5^C methylation. Unlike TETs, ALKBH1 initially converts m^5^C to the intermediate hm^5^C and further oxidizes hm^5^C to produce 5-formylcytosine, thus catalyzing m^5^C demethylation (30). (3) m^5^C readers: m^5^C-modified RNA within cells is predominately recognized and regulated by ALYREF and YBX1. ALYREF encourages the nuclear export of mRNAs, whilst YBX1 stabilizes mRNAs by binding to m^5^C-modified mRNAs via its cold shock domain. Overall, the m^5^C regulatory network entails sophisticated molecular mechanisms and is essential for the regulation of cell functions and gene expression (28, 31).

## 3 Role of RNA m^5^C methylation in HCC

Aberrant expression of m^5^C correlates to the onset, prognosis, and immune microenvironment of various cancers, such as lung cancer (32), head and neck squamous cell carcinoma (33), HCC (34), and gastrointestinal cancer (35). Prior studies reported the marked up-regulation of m^5^C methylation levels and related regulatory factors, including NSUN2-7, TRDMT1, TET1-3, and ALYREF in HCC tissues (36–41). Particularly, high NSUN2 expression has been identified as an independent risk factor for HCC as it has been tightly associated with immune cell infiltration, malignant progression, and poor prognosis in HCC (34, 42, 43). Prior research has highlighted that genes upregulated in m^5^C methylation are primarily involved in phosphokinase pathways such as RAS and PI3K-AKT pathways. Additionally, NSUN2 expression shares a positive correlation with the expression of numerous genes, such as GRB2, RNF115, AATF, ADAM15, RTN3, and HDGF. Real-time PCR in a former study revealed that NSUN2 down-regulation significantly diminished the mRNA expression of these related genes and regulated the RAS pathway, causing cell cycle arrest and then sensitizing HCC cells to sorafenib (36). Several studies showed that NSUN2 overexpression increased HCC cell proliferation by facilitating the interaction between H19 and G3BP1 and enhancing FZR1 mRNA stability (43, 44). Additionally, another study exhibited that NOP2 overexpression suppressed HCC cell proliferation, migration, and invasion by elevating m^5^C methylation of XPD (45). Importantly, the latest study of Gu et al. demonstrated that NSUN5 accelerated HCC cell proliferation through a ZBED3-dependent mechanism, providing a new therapeutic target for HCC (46). Accumulating studies also reported the role of m^5^C readers in HCC. For instance, Wang et al. observed that ALYREF knockdown altered the biological phenotypes of HCC cells, influenced the level of immune cell infiltration, and was correlated with the overall survival of patients (47). Xue et al. found an association of ALYREF overexpression and eukaryotic translation initiation factor 4A3 (eIF4A3) upregulation with poor prognosis in HCC (48). In addition, Ru et al. noted that YBX1 up-regulation contributed to immune evasion by up-regulating PD-L1, whilst YBX1 knockout reversed therapy resistance, implicating that targeting m^5^C regulators may enhance the efficacy of immunotherapy (49). Collectively, aberrant expression of m^5^C methylation and alterations in the related regulators impact the onset and progression of HCC at various levels, offering crucial clues for the understanding of molecular mechanisms underlying HCC and providing guidance for future research and treatment of HCC.

## 4 Potential regulatory role of RNA m^5^C methylation in innate immune pathways of HCC

The innate immune system is one of the initial lines of defense in the body and comprises various cells, proteins, and molecules that collaborate to combat various external threats. The innate immune system not only combats pathogens but also exerts an anti-tumor role (3, 50). Recent research has underscored the significance of m^5^C methylation, a pivotal post-transcriptional modification, in the mediation of RNA structure and function (8, 51). Specifically, m^5^C methylation modulates the stability and translation of specific RNAs, thereby activating innate immune pathways (14–16). Within the innate immune system, specialized receptors, such as TLRs (16) and cytosolic nucleic acid sensors like cGAS-STING (14) and RIG-I-like receptors (RLRs) (15), can recognize abnormal tumor nucleic acids. When activated, these receptors initiate immune responses, including inflammation and immune cell activation, to bolster the defense against tumors. A deeper comprehension of the interplay between m^5^C methylation and TLRs, cGAS-STING, and RLRs offers novel insights into mechanisms underlying the anti-tumor actions of the immune system.

### 4.1 The TLR pathway

TLRs, as key PRRs within the innate immune system, are responsible for recognizing invading pathogens, double-stranded RNA, single-stranded RNA, and CpG-DNA. Upon recognition, TLRs stimulate immune responses by activating intracellular pathways and transcription factors such as IRF7 and NF-κB via adapter proteins including MyD88 (7). There are 10 types of TLRs in the human liver, which are distributed on the cell surface and within intracellular compartments (52). Given the constant exposure of the liver to gut microbes, TLRs assume crucial roles in repressing inappropriate pro-inflammatory responses. On the contrary, prolonged TLR activation may elicit chronic liver damage and elevate the risk of HCC (53). Notably, diverse biological functions vary across TLRs. For instance, TLR4 activation can obviously promote the development of HCC by up-regulating pro-inflammatory factors and malignant tumor-related molecules and mediating immune cell infiltration (54–59). In contrast, TLR3 activation can protect against HCC cell growth by inducing reactive oxygen species generation and activating apoptotic pathways (60–62). Additionally, TLR2 (63), TLR5 (64), TLR7 (65, 66), TLR8 (66), and TLR9 (67) are also implicated in the development of HCC, whose activities may affect various processes such as cell proliferation, migration, and apoptosis.

**Figure 3:**
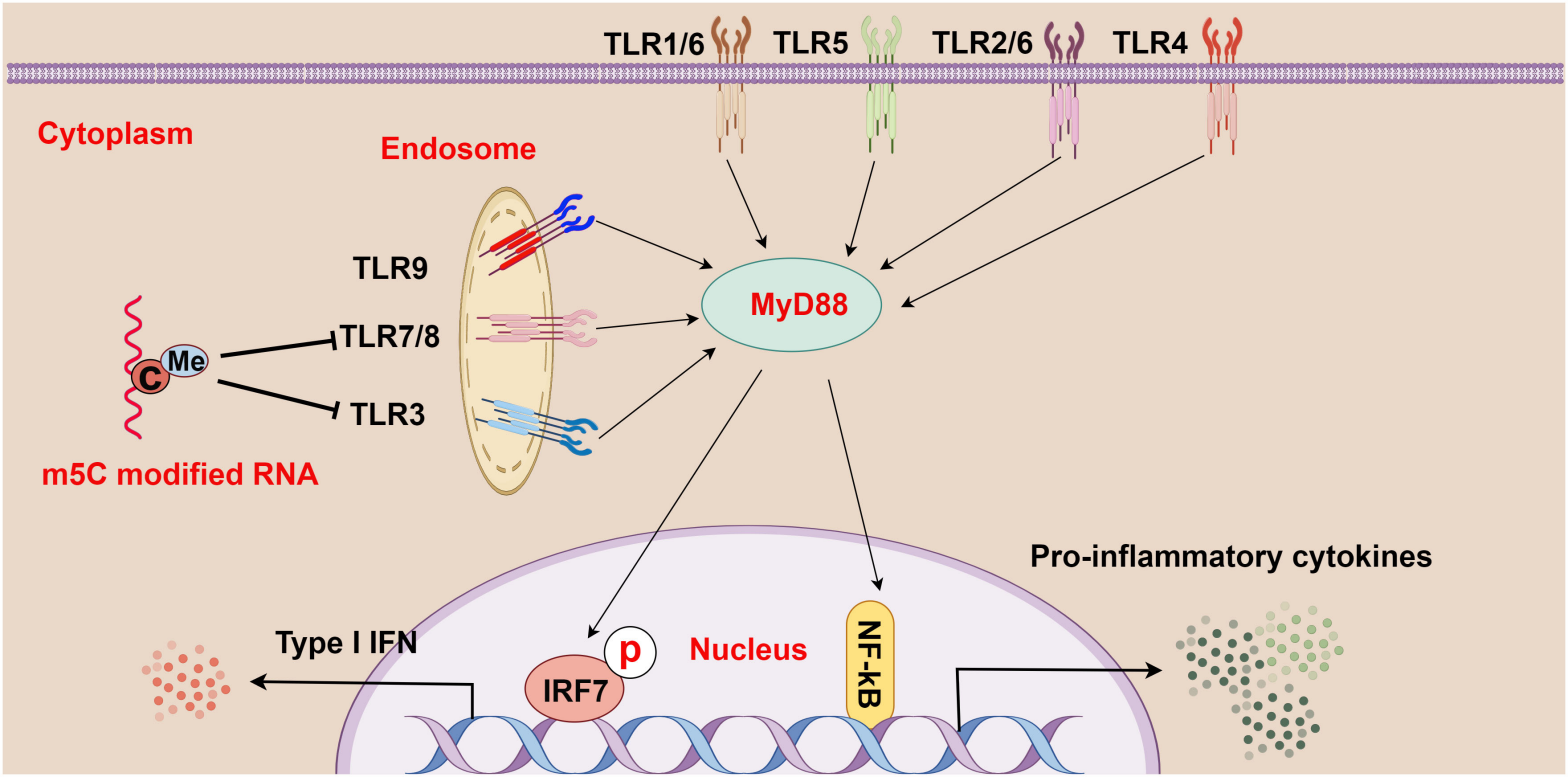
The relationship between m^5^C methylation and the Toll-like receptor (TLR) pathway (figure by Figdraw). TLR1-9 are ubiquitously distributed in cells, both on the cell surface and within internal organelles. Upon recognition of various pathogens and foreign molecules such as double-stranded RNA, single-stranded RNA, and CpG-DNA, these genes activate intracellular pathways and transcription factors (such as IRF7 and NF-κB), and these pathways signal through adapter proteins, such as MyD88. Moreover, m^5^C-modified RNA plays a crucial role in regulating this process by abrogating the signaling activity of TLR3, TLR7, and TLR8, thereby modulating the strength and scope of immune responses.

Through comparisons between m^5^C-modified RNA and unmodified RNA, Kariko et al. observed that m^5^C-modified RNA inhibited the expression of cytokines and activation markers in DCs and also suppressed the signaling activity activated by TLR3, TLR7, and TLR8, thereby affecting the immune activity of DC cells (16) (**Figure 3**). Furthermore, because base modification in mRNAs changes their immune activity, researchers used m^5^C in mRNA therapy alone or in combination with other modifications, which successfully inhibited the immune effects of TLR-mediated mRNAs (16). This breakthrough signifies a major advancement in the realms of RNA and immunotherapy. The research by Andries et al. further highlighted m^5^C-modified mRNAs as a novel mRNA therapy, partly because of their ability to boost evasion from the innate immune response by inhibiting endosomal TLR3 (68). In summary, m^5^C methylation may exert complex and critical effects on TLRs and the pathogenesis of HCC, offering essential insights into the immunological mechanisms of HCC and the development of treatment strategies for HCC.

### 4.2 The cGAS-STING pathway

As a cytoplasmic PRR and a DNA sensor, cGAS activates the IFN pathway by recognizing cytoplasmic DNA, including DNA released from viruses, bacteria, mitochondria, tumor cells, or dead cells. The C-terminus of cGAS contains a conserved zinc ion-binding region, which favors DNA binding and dimerization of cGAS. DNA binding changes the structure of cGAS, leading to cGAS activation. STING, a small protein in the endoplasmic reticulum, is retained in the endoplasmic reticulum through interactions with the Ca^2+^ sensor STIM1. When binding to DNA, cGAS is activated by ATP and GTP, catalyzing the formation of cGAMP, which further activates STING. Activated STING is palmitoylated and phosphorylated to recruit TBK1 and IKK, among which TBK1 triggers the release of IFN (a pivotal pathway in cancers, particularly HCC) by activating IRF3, while IKK induces inflammation by activating NF-κB (69, 70).

The involvement of the cGAS-STING pathway in HCC has been increasingly reported. For instance, a prior study revealed that olaparib activated STING chemokine signaling to enhance radiation-induced systemic anti-tumor effects (71). Another study showed that the cGAS-STING pathway was involved in the promoting effects of oncolytic influenza viruses combined with PD-L1 antibodies on CD8+ T cell activation (72). Su et al. found that TAK1 deficiency modulated ferroptosis and macrophage cGAS-STING signaling to foster liver injury and tumorigenesis (73). A study by Li et al. demonstrated that hyperbaric oxygen boosted teniposide-induced cGAS-STING activation, therefore increasing the anti-tumor efficacy of PD-1 antibodies in HCC (74). Gut microbiota was reported to modulate radiotherapy-induced anti-tumor immune responses in HCC via STING signaling (75). Sorafenib combined with STAT3 knockdown was revealed to accelerate HCC cell apoptosis and encourage cGAS-STING-mediated anti-tumor immunity (76). The disruption of the BRCA1-PALB2 interaction was demonstrated to induce tumor immunosuppression and T lymphocyte infiltration in HCC through the cGAS-STING pathway (77). According to the results of Du et al., radiotherapy induced PD-L1 up-regulation via cGAS-STING activation to facilitate immune evasion in HCC (78). Zhao et al. observed that hypoxia-induced RNASEH2A constrained cGAS-STING signaling activation in HCC and might portend the poor prognosis of patients (79). Of note, another study displayed that STING deficiency accelerated tumor growth and reduced autophagy and apoptosis in mice with HCC, which could be nullified by STING agonists.

**Figure 4:**
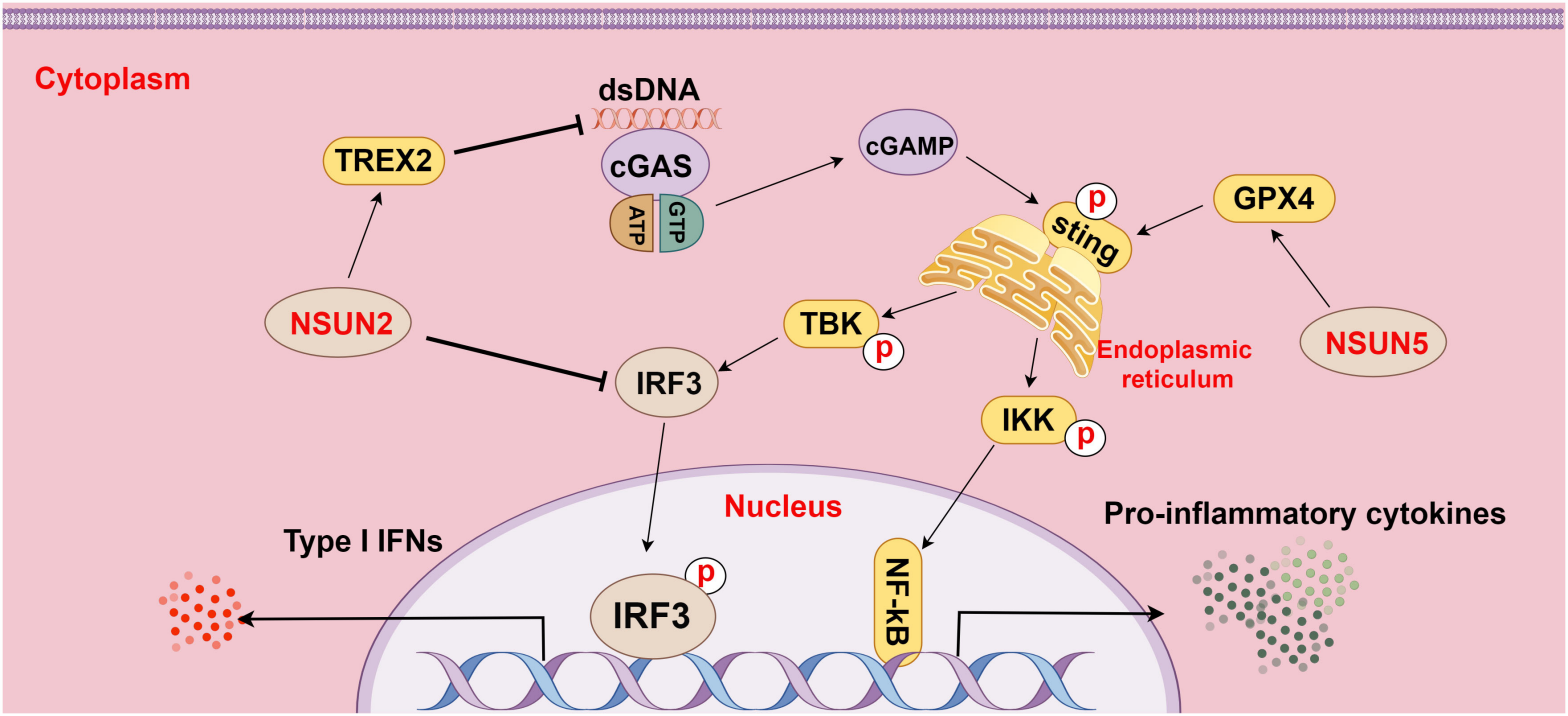
The relationship between m^5^C methylation and the cGAS-STING pathway (figure by Figdraw). cGAS is a cytoplasmic pattern recognition receptor and a DNA sensor, which activates the IFN pathway by recognizing double-stranded DNA (dsDNA) in the cytoplasm. NSUN2 inhibits dsDNA aggregation by methylating TREX2 mRNA and increasing TREX2 expression, thereby restraining the activation of the cGAS-STING pathway. Additionally, NSUN2 methylates IRF3 mRNA to facilitate IRF3 mRNA degradation and reduce IRF3 production, subsequently modulating the cGAS-STING pathway. Conversely, NSUN5 activates the cGAS-STING pathway by methylating GPX4 mRNA, thereby elevating the activity of the pathway.

Furthermore, the research of Chen et al. demonstrated that NSUN5-mediated m^5^C methylation of GPX4 contributed to cGAS-STING signaling activation, which fostered anti-cancer immune responses in colon adenocarcinoma (80). Another study revealed that NSUN2 activation maintained global m^5^C methylation of RNA, including TREX2, in tumor cells and stabilized TREX2 expression, consequently impeding the accumulation of cytoplasmic double-stranded DNA and blocking the activation of the cGAS-STING pathway, which facilitated tumorigenesis and PD-L1 immunotherapy resistance (14). Additionally, NSUN2-mediated m^5^C methylation of IRF3 mRNA may negatively affect type I IFN responses in various viral infections (81). These findings underscore the important effect of m^5^C methylation on the cGAS-STING pathway in HCC and highlight its potential as a hot spot for future research on HCC (**Figure 4**).

### 4.3 The RIG-I pathway

RLRs, a class of intracellular PRRs, are primarily tasked with the surveillance of RNA viruses, DNA viruses, and pathogenic RNA stemming from certain bacterial infections. Upon detection, RLRs lead to the activation of IRF3, a transcription factor, transcriptionally up-regulating cytokines such as IFNs and then bolstering immune responses. Mounting evidence supports the indispensable role of intact IFN signaling in the efficacy of numerous traditional chemotherapeutic drugs and targeted anti-cancer drugs. Accordingly, activation of RIG-I or MDA5 signaling has emerged as a promising therapeutic strategy for cancers (82, 83).

A prior study exhibited that hepatocyte-specific RIG-I deficiency facilitated diethylnitrosamine-induced hepatocarcinogenesis but mitigated non-alcoholic fatty liver disease-induced hepatocarcinogenesis. This study also demonstrated that interleukin-6 (IL-6) diminished RIG-I expression in HCC progenitor cells (HcPCs), driving IL-6 effector signaling and the development of HCC from HcPCs (84). NFATc3 was found to impede HCC and hepatitis B virus replication by positively regulating RIG-I-mediated IFN transcription, illustrating that NFATc3 may exert an effect on the RIG-I pathway, which is vital for maintaining liver health and repressing viral replication (85). In stem cells, RIG-I deficiency led to TGF-β/AKT/Smad2 signaling activation and precipitated the formation of tolerogenic DCs, underscoring the significance of RIG-I in immune regulation (86). Additionally, RIG-I has been identified not only as an anti-oncogene but also as a biomarker for the efficacy of IFN-α treatment for HCC, emphasizing its critical role in modulating the growth and treatment responses of tumors (6, 87).

**Figure 5:**
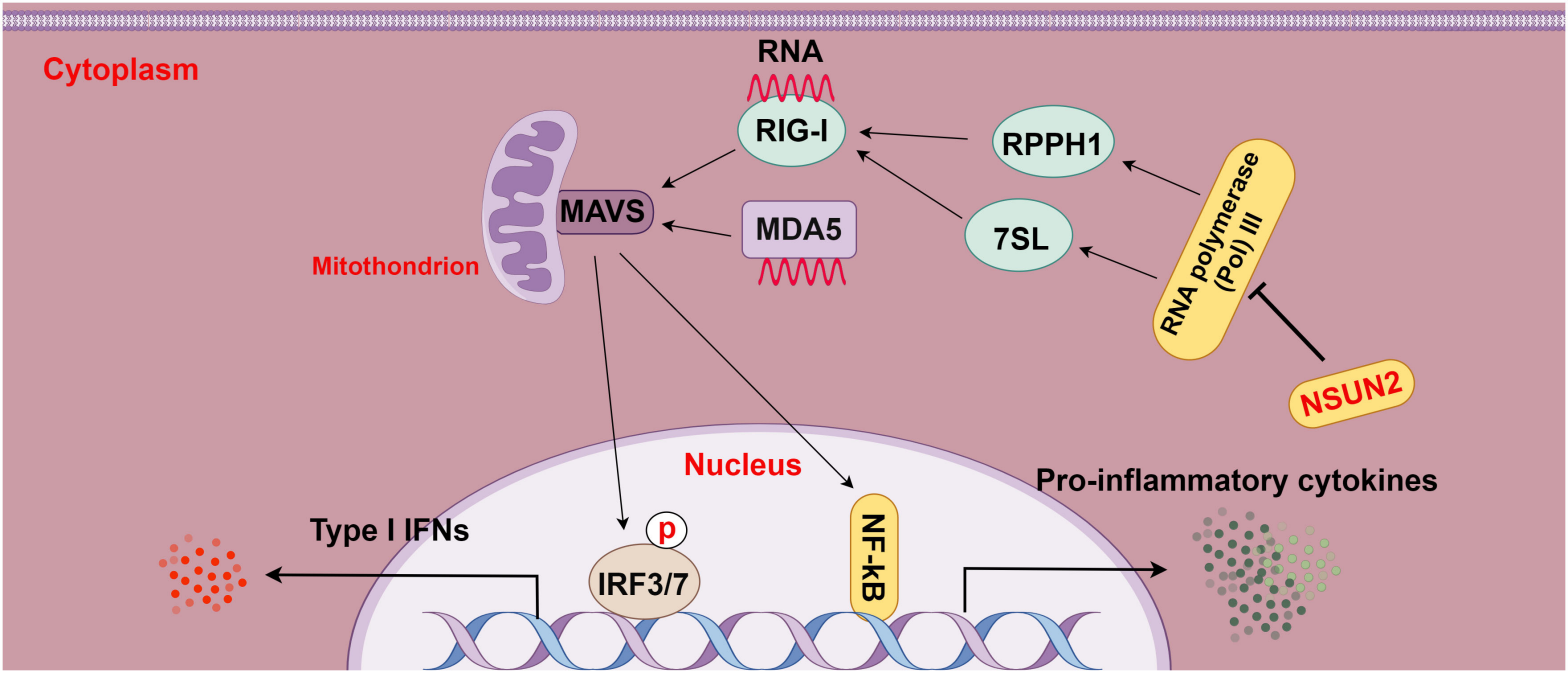
The relationship between m^5^C methylation and the RIG-I pathway (figure by Figdraw). RLRs, including RIG-I and MDA5, act as intracellular pattern recognition receptors (PRRs) that surveil RNA viruses, DNA viruses, and pathogenic RNA derived from tumors. Upon detection, RLRs activate the transcription factors IRF3/7, leading to the transcriptional up-regulation of interferons and other cytokines and then enhancing immune responses. NSUN2 mediates the RIG-I pathway by decreasing the transcription of non-coding RNAs regulated by host RNA polymerase III, particularly RPPH1 and 7SL RNA, ultimately suppressing RIG-I-mediated interferon responses, with no effect on MDA5 signaling.

Moreover, a prior study displayed that deletion of the m^5^C methyltransferase NSUN2 boosted type I IFN responses and substantially depressed the replication of various RNA and DNA viruses and that NSUN2 loss diminished the RNA m^5^C methyl group in the host and elevated the polymerase III transcription of non-coding RNAs recognized by RIG-I, particularly RPPH1 and 7SL RNA, consequently enhancing type I IFN signaling. In addition, this study also showed that this m^5^C-mediated antiviral innate immunity was also conserved in mouse models (15). Overall, this study elucidates the role of m^5^C in regulating innate immunity and illustrates m^5^C as a promising target for the development of broad-spectrum antiviral and anti-tumor therapeutics (**Figure 5**).

## 5 Challenges and prospects

Although great strides have been made in understanding the regulatory effect of m^5^C methylation on innate immune pathways, the role and mechanism of m^5^C methylation in different tumors remain poorly identified because tumor cells are highly heterogeneous. Various immune cells, including macrophages, DCs, NK cells, and neutrophils, can recognize distinct tumor-related antigen modifications which may involve alterations in cell metabolism and tissue anatomy. These alterations potentially serve as markers for tumor identification (3, 50, 88). Chemotherapy and radiotherapy have been observed to induce immunogenic cell death in tumors, resulting in the release of damage-associated molecular patterns such as cell surface calreticulin and heat shock proteins (3, 89), which can favor antigen presentation and secretion of immune stimulatory factors to activate innate immune cells. Additionally, cytokines including CXCL9, CXCL10, type I IFN, IL-2, and IL-15 can enhance the activity of immune cells, such as CD8 T and NK cells (90). Therefore, further studies are warranted to delve into the mechanisms by which m^5^C methylation influences the recognition of tumors by innate immunity, which is essential for advancing the understanding of tumor-immune interactions and the development of novel therapeutic strategies.

Current research on the role of m^5^C methylation in the innate immune pathways of HCC largely relies on RNA methylation sequencing technologies and bioinformatics analysis (28). Therefore, it is imperative to develop more precise high-throughput detection technologies, such as Nanopore Sequencing (91), Chemical Labeling Coupled with Mass Spectrometry (92), Single-Molecule Real-Time Sequencing (93), and Selective Chemical Labeling Followed by Sequencing (94), for accurately quantifying and localizing m^5^C methylation. With the ongoing advancements in nanomedicine technology (95, 96), we can anticipate the application prospects of nanocarriers in the regulation of immune pathways by m^5^C methylation. These nanocarriers may serve as vehicles for delivering RNA methylation-related drugs or RNA (96), enabling precise regulation of the immune microenvironment in HCC and thereby enhancing the efficacy of tumor immunotherapy. Conclusively, more studies are required to further explore the role of m^5^C methylation in the innate immune pathways of HCC, thus providing a novel theoretical foundation and clinical direction for the advancement and application of immunotherapy for HCC.

## Author contributions

SM: Conceptualization, Data curation, Formal analysis, Funding acquisition, Investigation, Methodology, Project administration, Resources, Software, Supervision, Validation, Visualization, Writing – original draft. BJ: Conceptualization, Data curation, Formal analysis, Funding acquisition, Investigation, Methodology, Project administration, Resources, Software, Supervision, Validation, Visualization, Writing – original draft. WH: Conceptualization, Data curation, Formal analysis, Funding acquisition, Investigation, Methodology, Project administration, Resources, Software, Supervision, Validation, Visualization, Writing – original draft. LM: Conceptualization, Data curation, Formal analysis, Funding acquisition, Investigation, Methodology, Project administration, Resources, Software, Supervision, Validation, Visualization, Writing – review & editing. ZL: Conceptualization, Data curation, Formal analysis, Funding acquisition, Investigation, Methodology, Project administration, Resources, Software, Supervision, Validation, Visualization, Writing – review & editing. LS: Writing – original draft, Writing – review & editing, Conceptualization, Data curation, Formal analysis, Funding acquisition, Investigation, Methodology, Project administration, Resources, Software, Supervision, Validation, Visualization.
